# Effects of Ground Glass Fiber on Late-Age Strength Evolution of Fly Ash–Cement Backfill Materials Under Prolonged CO_2_ Mineralization

**DOI:** 10.3390/ma19204358

**Published:** 2026-10-16

**Authors:** Luoyu Zhou, Shaoqi Kong, Ruixue Feng, Jiahao Gao, Zicheng Wei, Yi Zhou, Jiadong Guo

**Affiliations:** 1 College of Mining Engineering, Taiyuan University of Technology, Taiyuan 030024, China; 13892474348@163.com (L.Z.); 18079251850@163.com (J.G.); 18535029061@163.com (Z.W.); zy2024tyut@163.com (Y.Z.); 18536201839@163.com (J.G.); 2 College of Electrical and Power Engineering, Taiyuan University of Technology, Taiyuan 030024, China; fengruixue@tyut.edu.cn

**Keywords:** CO_2_ mineralization curing, ground glass fiber, fly ash–cement backfill material, late-age strength decline, pore structure, microstructural mechanism

## Abstract

**Highlights:**

- Under the investigated CO_2_-mineralization curing regime, a pronounced late-age strength decline was observed.
- Under CO_2_ mineralization, 1% GGF was associated with a 38.5% lower relative extent of 7–28 d strength decline than the unmodified mixture.
- Carbonate formation coexisted with lower porosity and larger median pore size.
- GGF increased strength under both conventional hydration and CO_2_ mineralization curing and improved late-age strength retention under the investigated CO_2_ curing condition.
- Strength enhancement was not simply governed by carbonate formation or global pore refinement.
- Local particle–matrix interactions may contribute to the observed strengthening, although the specific mechanisms require further verification.

**Abstract:**

Prolonged high-concentration CO_2_ mineralization may enhance early-age strength but cause a late-age strength decline in cementitious backfill materials. This study investigated how ground glass fiber (GGF) dosage (0%, 1%, and 2%) affects fly ash–cement backfill materials under conventional hydration and prolonged CO_2_ mineralization curing. Compressive strength was measured at 3, 7, and 28 d, together with X-ray diffraction, Fourier-transform infrared spectroscopy, thermogravimetric analysis, mercury intrusion porosimetry, and scanning electron microscopy with energy-dispersive spectroscopy. Under CO_2_ mineralization, the 0% GGF mixture decreased from 22.42 MPa at 7 d to 14.32 MPa at 28 d, indicating a 36.1% strength decline. Increasing GGF dosage to 1% increased the 28 d strength to 21.07 MPa and reduced the 7–28 d strength decline to 22.2%, whereas 2% GGF provided no further improvement. GGF also increased strength under conventional hydration, indicating a general strengthening effect together with improved late-age strength retention under CO_2_ curing. TGA showed increased carbonate-related mass loss after mineralization, but 1% GGF did not further increase it, while MIP showed lower total porosity but larger median pore size. These results indicate that the strength benefit of 1% GGF was not governed by greater carbonate formation or overall pore refinement and may involve local particle–matrix interactions.

## 1. Introduction

Amid the global transition toward low-carbon development, integrating industrial solid-waste utilization with carbon capture, utilization, and storage (CCUS) has attracted increasing attention as a strategy for improving resource efficiency and reducing environmental burdens. Mine backfilling can consume coal-based solid wastes while providing load-bearing support and contributing to goaf stabilization. Coupling backfilling with CO_2_ mineralization provides an opportunity to simultaneously utilize solid wastes, maintain structural support, and convert CO_2_ into carbonate-bearing products within the backfill system [1,2]. Recent studies have demonstrated that CO_2_-mineralized solid-waste-based backfill materials can achieve measurable CO_2_ sequestration while maintaining the mechanical requirements of backfill systems, and that fly ash itself can undergo mineralization-driven phase reconstruction that alters its cementitious activity [3,4]. From a practical perspective, CO_2_ mineralization curing therefore provides a material-level route for integrating CO_2_ utilization with waste-derived backfill materials. The process can be implemented under controlled curing conditions, although its effectiveness depends on factors such as CO_2_ transport, moisture availability, curing duration, and curing environment [5,6].

The mechanical response of cementitious materials to CO_2_ exposure depends strongly on the duration and curing conditions. Under suitable conditions, CO_2_ reacts with calcium-bearing phases to form CaCO_3_, which can contribute to early-age strength development and modify the pore structure. With continued CO_2_ exposure, however, decalcification and decomposition of calcium-(aluminum)-silicate-hydrate phases may lead to structural rearrangement and carbonation-induced shrinkage [7,8]. The carbonation behavior of C-S-H is also affected by its chemical composition and Ca/Si ratio, which can influence the interaction and transport of CO_2_ within the gel structure [9]. Consequently, the extent of carbonation alone may not be sufficient to characterize the long-term mechanical response of a cementitious material. For backfill materials subjected to prolonged high-concentration CO_2_ mineralization, an important scientific issue is therefore to clarify how the initial strength enhancement associated with carbonation evolves with curing time and how prolonged mineralization may be accompanied by a subsequent decline in mechanical performance.

Ground glass fiber (GGF) is of interest as a fine waste-derived modifier because its material characteristics are relevant to the microstructural evolution of cementitious matrices. Ground waste glass can function as a supplementary cementitious material or filler, with its performance influenced by particle size, composition, replacement level, and curing conditions [10,11]. Recent multiscale research has further shown that recycled GGF can affect hydration, interfacial bonding, pore structure, and mechanical properties of cement paste, highlighting the strong dependence of glass-fiber-derived materials on their interaction with the cementitious matrix [12]. These characteristics provide a basis for considering GGF as a potential modifier in fly ash–cement backfill materials. In addition, previous research has directly investigated ground glass fiber as a cementitious material [13]. More specifically, a recent study on a related fly ash–cement composite grouting system identified 1% GGF as a favorable dosage under conventional curing conditions [14]. Building on this evidence, 0% GGF was used as the reference condition, 1% GGF was selected as the primary modification level, and 2% GGF was included to examine whether increasing the dosage would provide further mechanical benefits or instead produce an unfavorable dosage effect.

Although GGF modification of fly ash–cement materials has been investigated under conventional curing conditions, the role of GGF during prolonged high-concentration CO_2_ mineralization requires further clarification. In particular, the mechanical benefit of GGF under prolonged mineralization cannot be interpreted solely from its general strengthening effect under conventional hydration. It remains important to distinguish whether the observed strength response is primarily associated with carbonate formation, changes in the global pore structure, or local interfacial and defect-related characteristics. This issue is particularly relevant because prolonged mineralization can simultaneously promote carbonate formation and alter calcium–silicate-based hydration products, potentially producing competing effects on strength retention. In addition, the chemical composition of waste-derived GGF varies among material sources, and its behavior should therefore be interpreted in relation to the specific GGF composition investigated in this study.

Based on these considerations, this study focuses on the late-age mechanical evolution of GGF-modified fly ash–cement backfill materials under prolonged high-concentration CO_2_ mineralization, with particular emphasis on the relationship between strength retention and multiscale microstructural evolution. It was hypothesized that, although GGF may provide a general strengthening effect under both curing environments, an appropriate dosage could also improve late-age strength retention during prolonged CO_2_ mineralization through changes in local microstructural and defect characteristics rather than simply through increased carbonate formation. Accordingly, this study investigates fly ash–cement composite backfill materials incorporating 0%, 1%, and 2% GGF under conventional hydration and prolonged high-concentration CO_2_ mineralization curing. Compressive strength was evaluated at 3, 7, and 28 d, while X-ray diffraction (XRD), Fourier-transform infrared spectroscopy (FTIR), thermogravimetric analysis (TGA), mercury intrusion porosimetry (MIP), and scanning electron microscopy coupled with energy-dispersive spectroscopy (SEM-EDS) were employed to characterize phase evolution, chemical-bond changes, thermal decomposition, pore structure, and local microstructural characteristics. Particular attention was given to three specific objectives: (i) characterizing how prolonged CO_2_ mineralization changes the strength evolution of fly ash–cement backfill materials; (ii) evaluating whether GGF improves late-age strength retention under prolonged mineralization; and (iii) examining whether the mechanical benefit of GGF is more closely associated with carbonate formation, global pore-structure evolution, or local interfacial and defect-related characteristics.

## 2. Materials and Methods

### 2.1. Raw Materials

Ordinary Portland cement (OPC), P.O 42.5 grade, manufactured by Tianrui Xin Deng Zhengzhou Cement Co., Ltd. (Zhengzhou, China), was used in this study. Its principal mineral phases were tricalcium silicate (C_3_S) and dicalcium silicate (C_2_S). The physical and chemical properties of the cement, including setting time and 3-day compressive strength, are listed in Table 1. Low-calcium fly ash (FA) was used as the supplementary cementitious material. Its major crystalline phases were mullite and quartz, and its chemical composition and technical properties are presented in Table 2. Ground glass fiber (GGF), supplied by Shijiazhuang Jiubao Building Materials Co., Ltd. (Shijiazhuang, China), was used as the micro-modifying material. The material was processed from recycled waste continuous glass fibers and milled to a nominal fineness of 400 mesh, corresponding to an equivalent particle size of approximately 38 μm. The chemical composition of the GGF, as determined by X-ray fluorescence (XRF) spectrometry, is detailed in Table 2, with CaO (52.43%) and SiO_2_ (45.89%) being the principal oxides.

Six experimental groups were designed to investigate the effects of GGF dosage and curing regime. Cement and fly ash were proportioned at a mass ratio of 70:30 and collectively defined as the base binder. GGF was incorporated as an additional component at 0%, 1%, and 2% by mass of the base binder. The water content was fixed at 45 parts per 100 parts of base binder, corresponding to a water-to-base-binder mass ratio of 0.45. The detailed mix proportions are presented in Table 3. Each mixing batch weighed approximately 3.5 kg and yielded nine cylindrical specimens, with three specimens tested at each curing age of 3, 7, and 28 d.

### 2.2. Specimen Preparation and Curing

During mixing, the weighed cement, fly ash, and GGF were first dry-mixed uniformly. A predetermined amount of water was then added. The slurry was mixed at a low speed for 2 min and then at a high speed for 2 min until a homogeneous mixture was obtained. The slurry was cast into cylindrical molds with a diameter of 50 mm and a height of 100 mm, compacted on a vibration table, and covered with a thin film to minimize water evaporation.

**Figure 1:**
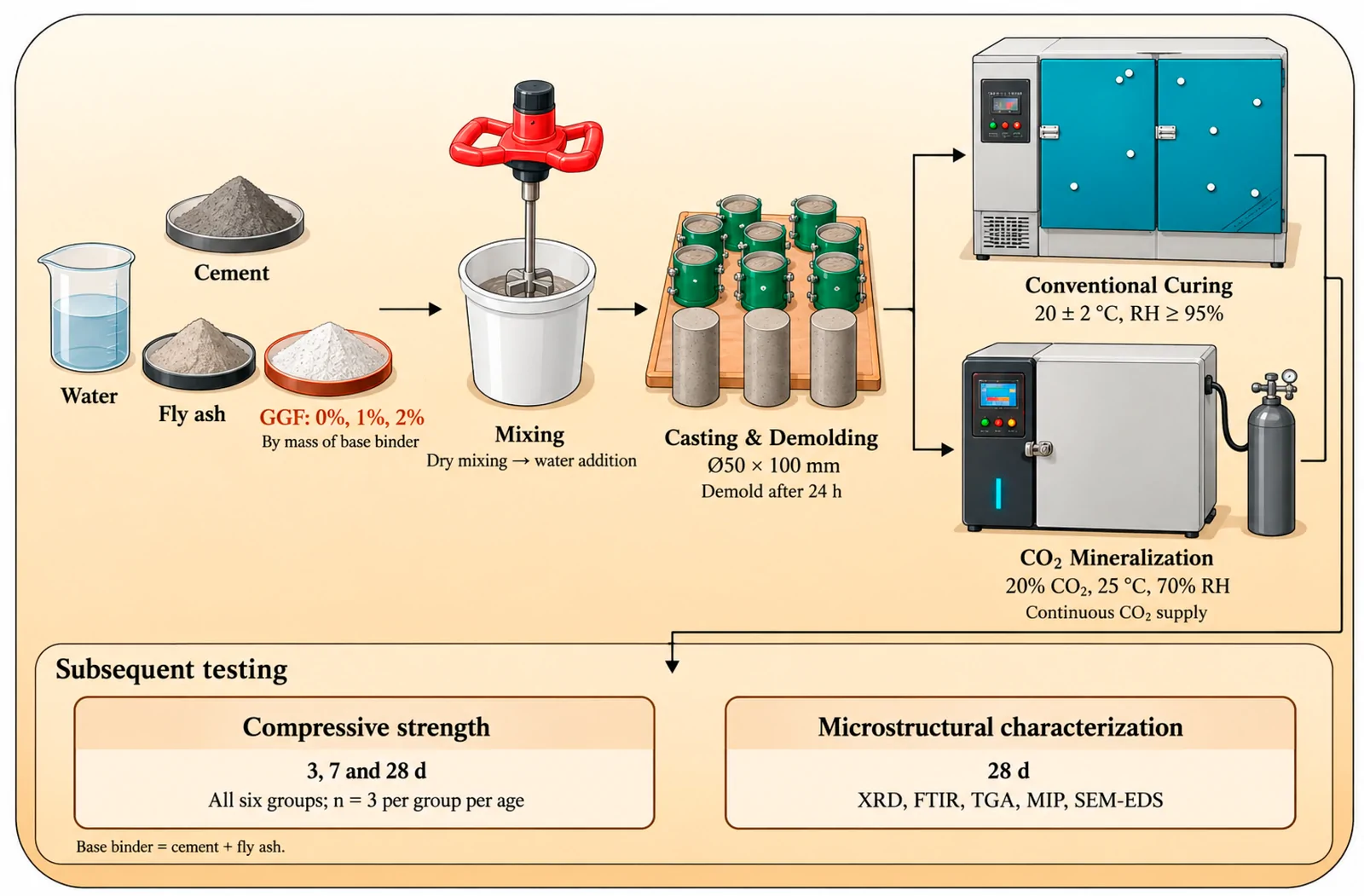
Schematic illustration of specimen preparation, GGF addition, curing regimes, and subsequent testing.

After standing at room conditions for 24 h, the specimens were demolded and subjected to two distinct curing regimes (Figure 1). For conventional hydration curing, the C-series specimens were placed in a standard curing chamber maintained at 20 ± 2 °C with a relative humidity (RH) of ≥95% until the designated curing ages. For CO_2_ mineralization curing, the G-series specimens were placed in a carbonation curing chamber with a controlled CO_2_ concentration of 20%, an RH of 70%, and a temperature of 25 °C. CO_2_ was continuously supplied until the testing ages of 3, 7, and 28 days. Previous studies have shown that carbonation progression in cementitious materials is strongly influenced by relative humidity and curing conditions [15].

Because the conventional hydration and CO_2_ mineralization curing regimes differed in both CO_2_ concentration and temperature/relative humidity, comparisons between the two regimes in subsequent sections are interpreted as differences between the overall curing environments rather than as isolated effects of CO_2_ concentration alone.

### 2.3. Testing and Characterization Methods

#### 2.3.1. Compressive Strength Test

Cylindrical specimens cured for 3, 7, and 28 d were subjected to uniaxial compressive strength testing using a microcomputer-controlled electronic universal testing machine (WDW-100, Xuzhou Dongkong Technology Co., Ltd., Xuzhou, China) with a maximum load capacity of 100 kN. The test procedure was conducted with reference to GB/T 23561.7-2009 [16], Methods for Determining the Physical and Mechanical Properties of Coal and Rock—Part 7: Methods for Determining the Uniaxial Compressive Strength and Counting Softening Coefficient. The specimens were loaded axially under displacement control at a rate of 0.5 mm/min until failure. Three independent specimens were tested for each mixture and curing age (*n* = 3). Compressive strength was calculated from the maximum failure load divided by the original cross-sectional area of the specimen. The results were expressed as the mean ± standard deviation (SD).

#### 2.3.2. Microstructural Characterization

**Figure 2:**
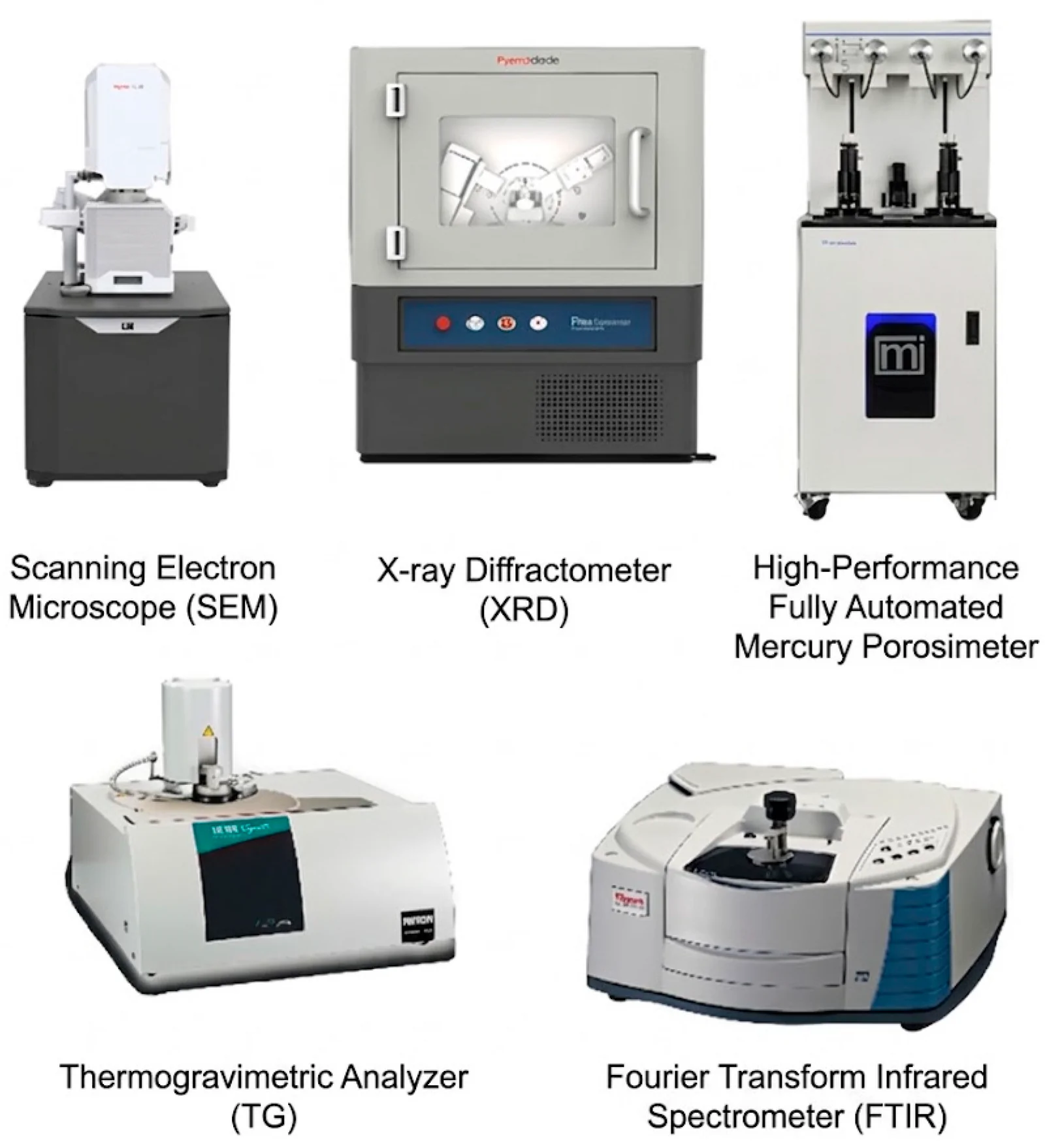
Experimental equipment.

The principal equipment used for microstructural characterization is shown in Figure 2. One representative specimen from each of the C0, C1, G0, and G1 groups at 28 d was selected for microstructural characterization. Since the macroscopic mechanical results indicated that 1% GGF provided the most favorable late-age mechanical performance within the investigated dosage range, the microstructural investigation was specifically focused on the 0% and 1% groups. This targeted selection was designed to compare the microstructural characteristics of the unmodified and 1% GGF-modified materials under the two curing regimes and to explore possible microstructural factors associated with the observed mechanical response, while the less mechanically effective 2% dosage group was excluded from extensive micro-level analysis. Because sample pretreatment can affect MIP measurements, the samples were collected from the interior of the specimens [17]. Samples for XRD, FTIR, and TGA were ground into powder, whereas block specimens were used for MIP and SEM-EDS, with SEM observations performed on fracture surfaces. Hydration was terminated using anhydrous ethanol, followed by vacuum drying to constant mass before characterization. Because fracture-surface preparation and vacuum drying may generate or enlarge crack-like features, cracks observed by SEM were interpreted qualitatively and were not assumed to represent pre-existing in situ microcracks. Their possible relation to material deterioration was considered only in conjunction with the independent mechanical, MIP, XRD, FTIR, and TGA results.

X-ray diffraction (XRD): A Rigaku SmartLab SE X-ray diffractometer (Rigaku Electric Co., Ltd., Tokyo, Japan) was used to characterize phase evolution. The scanning range was 5–90°, with a scanning rate of 2°/min.

Thermogravimetric analysis (TGA): A HITACHI STA200 simultaneous thermal analyzer (HITACHI, Tokyo, Japan) was used to characterize the thermal decomposition behavior associated with hydration and carbonation products. The samples were heated from room temperature to 1000 °C under a pure N_2_ atmosphere at a heating rate of 10 °C/min.

Fourier-transform infrared spectroscopy (FTIR): A Thermo Scientific Nicolet iS20 spectrometer (Thermo Fisher Scientific, Waltham, MA, USA) was used. Samples were prepared via the KBr pellet method and analyzed over a wavenumber range of 400–4000 cm^−1^ with a resolution of 4 cm^−1^ and 32 scans.

Mercury intrusion porosimetry (MIP): An automated high-performance mercury porosimeter (Micromeritics AutoPore V 9605, Micromeritics Instrument Corporation, Norcross, GA, USA) was used, assuming a mercury contact angle of 130°, covering a pore-size range of 0.005–340 μm.

Scanning electron microscopy coupled with energy-dispersive spectroscopy (SEM-EDS): A ZEISS Sigma 300 field-emission SEM (Carl Zeiss AG, Oberkochen, Germany) operated at an accelerating voltage adjusted between 5 and 15 kV was used to observe fracture morphologies. EDS was simultaneously employed for localized elemental analysis. Specimen surfaces were gold-coated prior to observation to enhance conductivity.

#### 2.3.3. Data Processing

Compressive strength results are reported as the mean ± sample standard deviation (SD) of three independent specimens. At each curing age, differences among GGF dosages were analyzed separately within each curing regime using one-way analysis of variance (ANOVA), followed by Tukey’s honestly significant difference (HSD) test for pairwise comparisons. Statistical significance was set at *p* < 0.05. All statistical analyses were performed using SciPy (version 1.17.0). XRD patterns were used for qualitative phase identification and comparison of phase evolution, and normalized peak intensities were not interpreted as quantitative phase contents. TGA data were analyzed according to the mass-loss ranges associated with the relevant thermal decomposition processes. FTIR spectra were compared based on changes in the positions and intensities of characteristic absorption bands. MIP data were used to determine total porosity, pore-size distribution, cumulative pore volume, and median pore diameter. SEM observations were interpreted qualitatively, while EDS was used for localized semiquantitative elemental analysis.

## 3. Results and Discussion

### 3.1. Mechanical Strength Evolution

**Figure 3:**
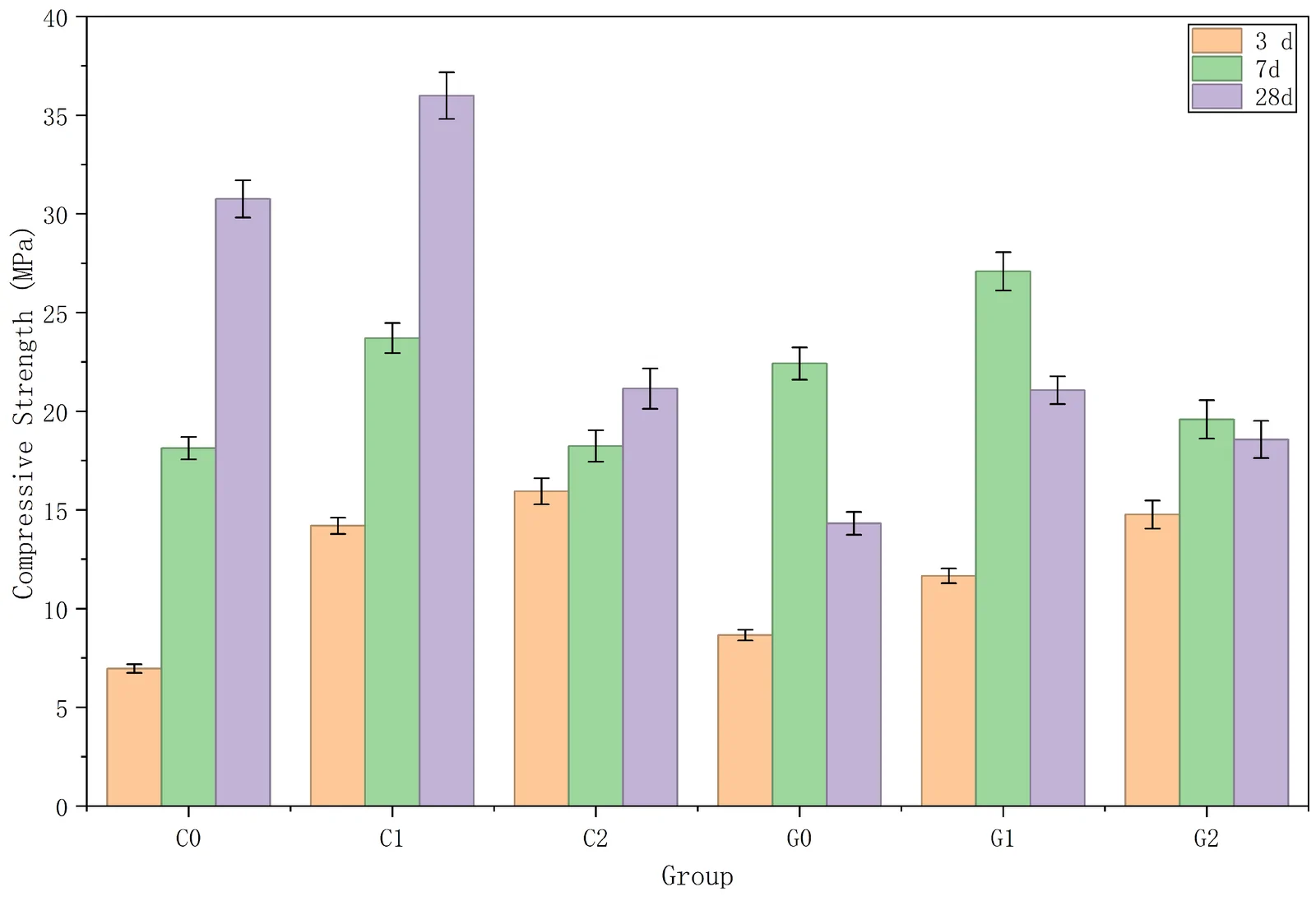
Uniaxial compressive strength at different curing ages under conventional hydration and CO_2_ mineralization curing (error bars represent the standard deviation of three replicates).

As shown in Figure 3, where error bars represent the standard deviation of three replicates, the compressive strength of all specimens under conventional hydration curing generally increased with curing age. At the evaluated curing ages, one-way ANOVA indicated significant effects of GGF dosage on compressive strength within the conventional-curing series (*p* < 0.05). For the unmodified C0 group, the compressive strengths at 3, 7, and 28 d were 6.96 (±0.22), 18.13 (±0.57), and 30.76 (±0.94) MPa, respectively. The 28 d compressive strength of the 1% GGF-modified C1 group reached 35.99 (±1.18) MPa, representing a statistically significant increase of 17.0% compared with C0. These results demonstrate that GGF addition increased the compressive strength under conventional hydration curing. Although fine glass powders have been reported to exhibit filler and pozzolanic effects [11], the present study did not directly quantify the reaction degree of GGF; therefore, the observed strength increase is not attributed to a specific pozzolanic reaction.

In contrast to conventional hydration curing, the specimens subjected to CO_2_ mineralization exhibited a distinct stage-dependent strength evolution. The compressive strength of G0 reached 22.42 (±0.82) MPa at 7 d but decreased to 14.32 (±0.58) MPa at 28 d. With the incorporation of 1% GGF, the compressive strengths of G1 at 7 and 28 d significantly improved to 27.09 (±0.97) and 21.07 (±0.70) MPa, respectively (*p* < 0.05).

To quantitatively characterize the extent of late-age strength decline under mineralization curing, the strength decline ratio *R_s_* was defined as:

(1) $$R_{s}=\frac{\sigma_{7d}-\sigma_{28d}}{\sigma_{7d}}\times 100\%$$

where *σ*_7_*_d_* and *σ*_28_*_d_* are the uniaxial compressive strengths of the specimens at 7 and 28 d, respectively (MPa).

According to Equation (1), the 7–28 d strength decline ratio of G0 was 36.1%, indicating pronounced late-age strength loss under the 20% CO_2_ mineralization condition adopted in this study. The corresponding value for G1 was 22.2%, which was 13.9 percentage points lower than that of G0.

To further quantify the difference in the 7–28 d strength decline between G0 and G1, a relative reduction index for the strength-decline ratio *η* was defined using G0 as the reference:

(2) $$\eta \;=\;\frac{R_{s,G0}\;-\;R_{s,G1}}{R_{s,G0}}\;\times \;100\%$$

where *R_s_*_,_*_G_*_0_ and *R_s_*_,_*_G_*_1_ are the strength decline ratios of the G0 and G1 groups, respectively.

According to Equation (2), *η* was 38.5%, indicating that the 1% GGF mixture showed a 38.5% lower relative extent of 7–28 d strength decline than G0. In addition, the 28 d compressive strength of G1 was 47.1% higher than that of G0.

It is important to distinguish between a general strengthening effect of GGF and a carbonation-specific mitigation effect. At 28 d, the incorporation of 1% GGF increased the compressive strength from 30.76 to 35.99 MPa under conventional hydration curing, corresponding to an absolute increase of 5.23 MPa. Under CO_2_ mineralization curing, the corresponding increase was from 14.32 to 21.07 MPa, i.e., 6.75 MPa. Although the relative strength increase was larger under CO_2_-mineralization curing because of the lower G0 reference strength, the absolute strength gains differed by only 1.52 MPa between the two curing regimes. Therefore, the present strength data demonstrate that 1% GGF improves strength under both curing regimes and is associated with a smaller 7–28 d strength decline under CO_2_ mineralization. However, these data alone do not establish whether GGF provides a distinct carbonation-specific mitigation effect beyond its general strengthening effect. Demonstration of such an interaction would require additional statistical or mechanistic evidence specifically separating the effects of GGF addition and curing regime.

A non-monotonic, age-dependent strength response was observed with increasing GGF dosage. At 3 d, the 2% GGF mixtures exhibited higher compressive strengths than the corresponding 1% mixtures under both curing regimes. However, this early-age advantage was not sustained. Under CO_2_ mineralization curing, G2 exhibited a compressive strength of 19.59 ± 0.97 MPa at 7 d, lower than those of G0 (22.42 ± 0.82 MPa) and G1 (27.09 ± 0.97 MPa). At 28 d, G2 reached 18.57 ± 0.94 MPa, representing a 29.7% increase relative to G0 (14.32 ± 0.58 MPa), but remained below G1 (21.07 ± 0.70 MPa). These results demonstrate that 2% GGF still improved the absolute 28 d compressive strength relative to the unmodified mixture, although the improvement was less pronounced than that achieved with 1% GGF. Notably, the relative 7–28 d strength decline was 5.2% for G2, compared with 22.2% for G1 and 36.1% for G0. The smaller relative decline of G2 should be interpreted in conjunction with its lower 7 d strength rather than as definitive evidence of greater resistance to mineralization-induced deterioration. Thus, 1% GGF provided the highest absolute 28 d compressive strength among the investigated dosages, whereas 2% GGF exhibited the smallest relative late-age strength decline.

The non-monotonic dosage response suggests that the mechanical benefits of GGF incorporation do not increase proportionally with its content. In the present mixture design, GGF was added while the cement, fly ash, and water contents were maintained constant. Increasing the GGF dosage may therefore affect particle packing, dispersion, and local particle–matrix interactions, potentially altering the balance between beneficial and unfavorable microstructural effects. Notably, C2 also exhibited a lower 28 d compressive strength than C1 under conventional hydration curing, indicating that the reduction in strength associated with increasing GGF from 1% to 2% was not exclusive to the CO_2_ mineralization environment. A similar non-monotonic dosage-dependent response has been reported for glass fiber powder-modified fly ash–cement grouting materials under conventional curing conditions [14]. Nevertheless, because G2 was not included in the microstructural characterization, the contributions of particle dispersion, interfacial heterogeneity, and hydration- or carbonation-related transformations cannot be distinguished from the present evidence. Accordingly, these factors are considered plausible explanations for the observed dosage dependence rather than experimentally verified mechanisms.

Therefore, within the investigated GGF dosage range of 0–2%, 1% GGF was identified as the most favorable tested dosage based on the highest absolute 28 d compressive strength under CO_2_ mineralization curing. This criterion should be distinguished from the relative 7–28 d strength decline, and the resulting dosage selection should not be interpreted as a universal optimum across different curing conditions or performance criteria.

### 3.2. Phase Evolution and Carbonation Behavior (XRD and TGA)

**Figure 4:**
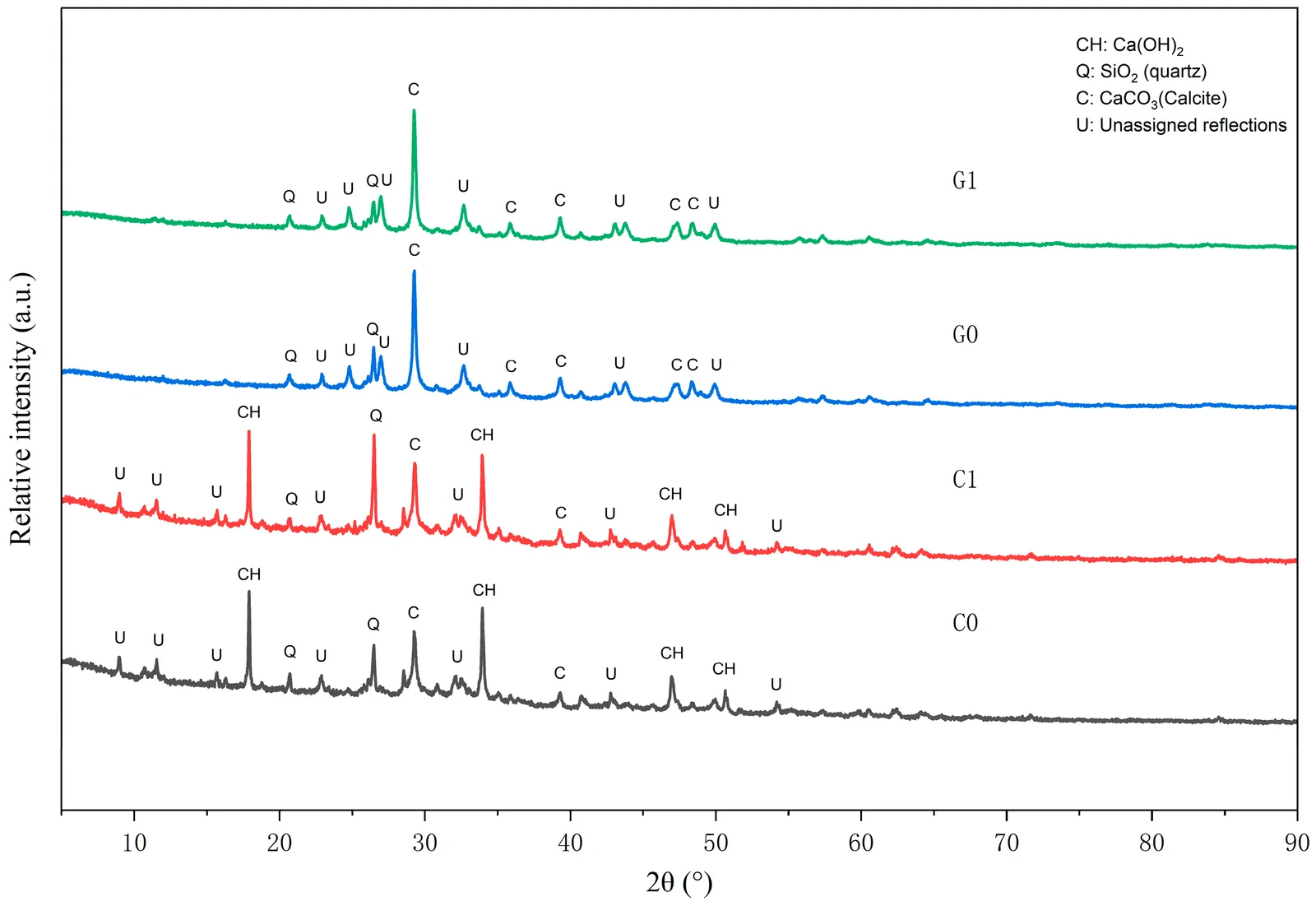
XRD patterns of different specimens at 28 d.

XRD and TGA were used to examine the phase assemblages and thermal decomposition characteristics associated with the observed strength evolution. Figure 4 presents the XRD patterns of C0, C1, G0, and G1 at 28 d. In the conventionally hydrated specimens, C0 and C1, portlandite reflections were observed near 2θ = 17.9° and 34.0°. These reflections were substantially weakened or no longer clearly resolved in G0 and G1 after CO_2_ mineralization curing, whereas the calcite reflection near 29.3° became more prominent. This change is consistent with the consumption of calcium-bearing phases and the formation of carbonate products during mineralization [18,19], in agreement with the progressive structural changes reported for hardened silicate cement systems exposed to CO_2_ [20].

In C0 and C1, the weaker reflections near 2θ = 9.0° and 15.7° are consistent with ettringite, which may also contribute to the feature near 22.8°, where overlap with calcite is possible [21]. A portlandite contribution to the feature near 54.2° is supported by the accompanying reflections near 17.9°, 33.9°, 47.0°, and 50.6°. The feature near 11.5° may contain contributions from gypsum or monocarboaluminate-type AFm, whose relevant reflections occur at similar positions [22]. Residual calcium silicate clinker phases may also contribute to the weak features between 32.1° and 32.5°. These interpretations provide possible assignments for several reflections marked U, although some features, including that near 42.8°, remain unresolved.

In G0 and G1, the combination of reflections near 2θ = 24.8°, 27.0°, 32.7°, and 43.8°, together with the feature near 49.9°, is consistent with the reported diffraction pattern of vaterite [23]. This correspondence across multiple reflections supports a probable vaterite contribution in both mineralized specimens, although the feature near 49.9° may also contain a quartz contribution. Calcite is supported by the prominent reflection near 29.3° and accompanying reflections near 35.9°, 39.3°, 47.4°, and 48.4°, with possible additional contributions near 22.9° and 43.0°. The mineralized patterns thus support the presence of calcite together with a tentative vaterite contribution. The U notation indicates that the proposed assignments remain uncertain where individual phase contributions cannot be distinguished reliably.

G0 and G1 exhibit broadly similar identifiable crystalline phase assemblages, with differences mainly in relative peak prominence, including the quartz-containing region near 26.4°. Because the patterns were normalized and no quantitative phase refinement was performed, these differences cannot establish phase contents or the extent of CO_2_ mineralization. The higher 28 d compressive strength of G1 (21.07 MPa) relative to G0 (14.32 MPa) is not accompanied by an additional set of well-resolved reflections. The XRD results therefore do not identify a new major crystalline product that accounts for the strength advantage of G1. Differences in poorly crystalline binding phases, reaction-product distribution, pore structure, and local particle–matrix interactions may also contribute to the mechanical response and are considered alongside the TGA, FTIR, MIP, and SEM-EDS results.

**Figure 5:**
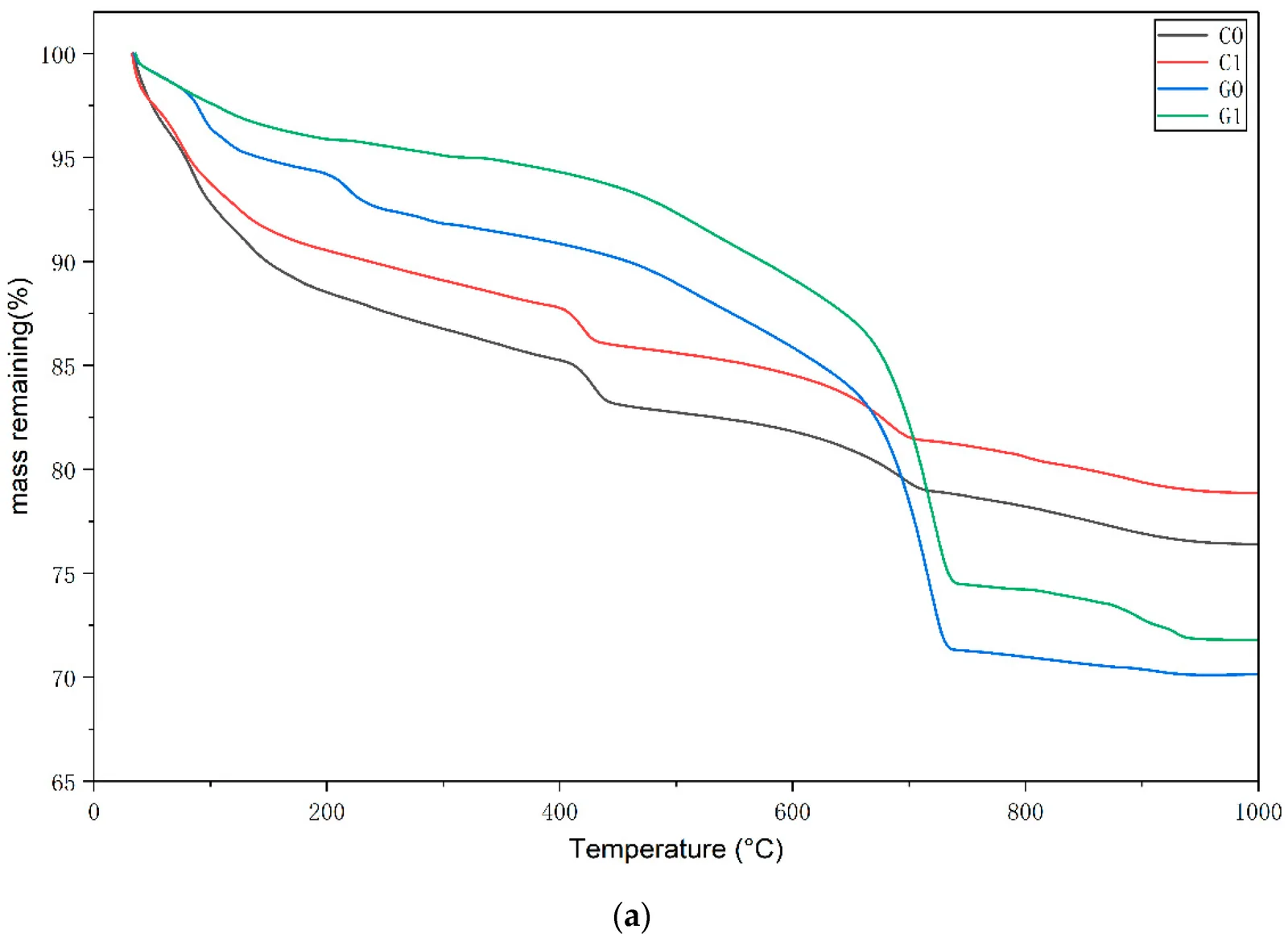

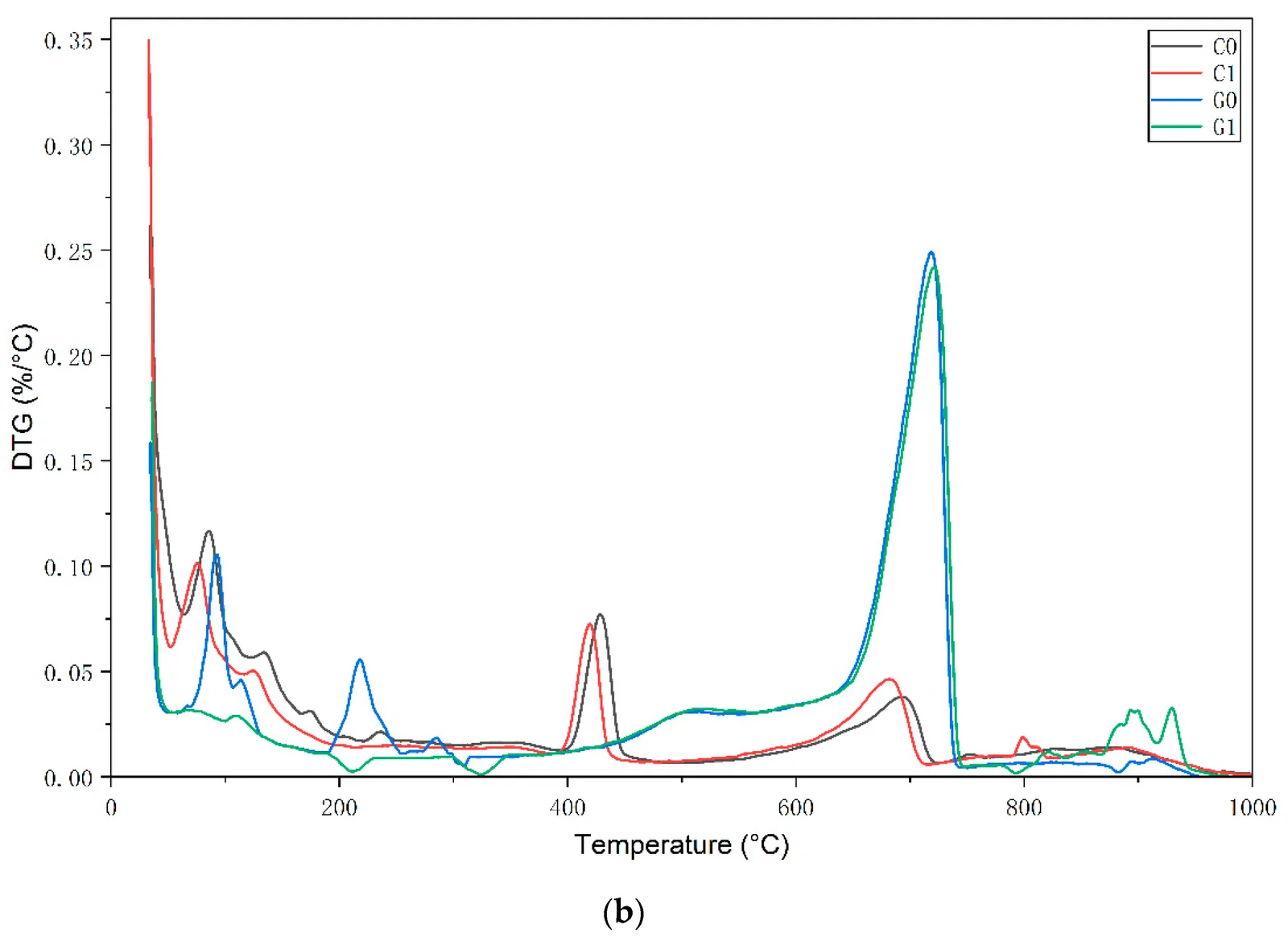
TGA and DTG curves of different specimens: (**a**) TG curves; (**b**) DTG curves.

TGA results further revealed differences in thermal decomposition behavior between specimens subjected to the two curing regimes (Figure 5). In the lower-temperature region (approximately 50–300 °C), mass loss is mainly associated with the removal of physically adsorbed and weakly bound water and the overlapping dehydration of hydration products, including C–(A)–S–H-type gels and Al-bearing hydrates such as AFt/AFm phases. Because these thermal events overlap, individual DTG features cannot be uniquely assigned to specific hydration phases. At approximately 400–500 °C, the Ca(OH)_2_-related dehydroxylation feature was markedly weakened or nearly absent in G0 and G1, consistent with the XRD results [19]. Both mineralized specimens exhibited pronounced carbonate-related decomposition features between 600 and 800 °C, with the main DTG peak near 700 °C [18]. The reported mass losses within this interval were 15.61% for G0, 15.36% for G1, and 4.39% for the conventionally cured C1 specimen. In particular, the comparison between G1 and C1, which contained the same GGF dosage, indicates substantially greater carbonate-related mass loss under the mineralization-curing environment.

A more detailed comparison revealed distinct differences between G0 and G1 in the lower-temperature region. G0 exhibited relatively pronounced DTG features at approximately 92 and 219 °C, whereas the corresponding features were substantially weaker or less clearly resolved in G1. Based on the original TG data, the mass losses of G0 and G1 within 50–150 °C were 4.26% and 2.63%, respectively, while those within 150–300 °C were 3.05% and 1.39%. Consequently, the cumulative mass losses within 50–300 °C were 7.32% for G0 and 4.02% for G1. A similar trend was observed under conventional hydration curing, where the corresponding mass losses were 10.73% for C0 and 8.52% for C1. This indicates that the lower low-temperature mass loss associated with GGF incorporation was not exclusive to CO_2_ mineralization curing.

These differences may reflect changes in hydration products during initial hydration and subsequent CO_2_ mineralization. During the initial hydration period, the cement–fly ash binder forms C–(A)–S–H-type gels, portlandite, and Al-bearing hydrates. Following demolding at 24 h, continued hydration and CO_2_-induced reactions may occur concurrently in the mineralized specimens. Importantly, carbonation in the fly ash–cement system is not limited to the reaction between portlandite and CO_2_. Calcium-bearing hydration products, including those formed with the participation of fly ash, may undergo progressive decalcification and carbonation during prolonged CO_2_ exposure, potentially producing carbonate phases and more Si/Al-rich residual structures [7,8]. These transformations may alter the composition, water-binding characteristics, and thermal response of the residual hydration products. Accordingly, the weaker low-temperature DTG features of G1 may reflect differences in the distribution or thermal behavior of these products. However, the overlapping dehydration processes prevent direct identification of individual phase changes or confirmation that GGF enhances hydration or suppresses C–(A)–S–H decalcification.

GGF incorporation may further modify the local physical and interfacial environments in which hydration and carbonation products develop. Nevertheless, the similar carbonate-related mass losses of G0 and G1 within 600–800 °C do not indicate an appreciable increase in the principal carbonate-decomposition signal following GGF incorporation. These results do not independently establish the overall degree of carbonation or demonstrate a distinct pozzolanic reaction or GGF-induced carbonation mechanism. Therefore, the observed TG/DTG differences are more conservatively interpreted as possible changes in the characteristics or thermal responses of hydration and carbonation products. The relative contributions of these processes cannot be quantitatively separated using the present TG/DTG results alone, and their potential relationships with the improved mechanical performance of G1 require consideration alongside the FTIR, MIP, and SEM-EDS observations.

Assuming that carbonate decomposition was the dominant contributor to mass loss between 600 and 800 °C, the corresponding TGA-derived CO_2_-equivalent mass fraction was estimated according to the corresponding stoichiometric relationship:

(3) $$\omega_{{\mathrm{CO}}_{2},\mathrm{TGA}}\approx \Delta m_{600–800}$$

where $\omega_{{\mathrm{CO}}_{2},\mathrm{TGA}}$ is the CO_2_ mass fraction estimated from TGA (%), and Δ*m*_600–800_ is the mass loss within the 600–800 °C temperature range (%).

The equivalent CaCO_3_ content was further calculated according to the stoichiometric relationship:

(4) $${\omega_{{\mathrm{CaCO}}_{3}}}^{\mathrm{eq}}\;\approx \;{\Delta m}_{600–800}\;\times \;\frac{{\mathrm{MCaCO}}_{3}}{{\mathrm{MCO}}_{2}}$$

where ${\omega_{{\mathrm{CaCO}}_{3}}}^{\mathrm{eq}}$ is the equivalent mass fraction of CaCO_3_ (%), and *MCaCO*_3_ and *MCO*_2_ are the molar masses of CaCO_3_ and CO_2_, respectively, taken as 100.09 and 44.01 g/mol.

According to Equations (3) and (4), the 4.39% mass loss of C1 in the 600–800 °C range corresponds to an estimated CO_2_ amount of approximately 43.9 mg/g and an equivalent CaCO_3_ content of approximately 9.98 wt%. For G0, the corresponding values were approximately 156.1 mg/g and 35.50 wt%, respectively, based on a mass loss of 15.61%. For G1, the estimated CO_2_ amount and equivalent CaCO_3_ content were approximately 153.6 mg/g and 34.93 wt%, respectively, corresponding to a mass loss of 15.36%.

To characterize the increase in carbonate decomposition-related mass loss in the mineralized groups relative to the conventionally cured C1 group, the following parameter was defined:

(5) $${\Delta \omega }_{{\mathrm{CO}}_{2},\mathrm{TGA}}\approx {\Delta m}_{600–800,G}-{\Delta m}_{600–800,C1}$$

where ${\Delta \omega }_{{\mathrm{CO}}_{2},\mathrm{TGA}}$ is the increase in the TGA-estimated CO_2_ amount in the mineralized group relative to C1 (%), and Δ*m*_600–800,_*_G_* and Δ*m*_600–800,_*_C_*_1_ are the mass losses of the mineralized group and C1 in the 600–800 °C range, respectively.

According to Equation (5), the increases in carbonate-related mass loss for G0 and G1 relative to C1 were 11.22 and 10.97 percentage points, corresponding to approximately 112.2 and 109.7 mg/g, respectively. These values characterize the increase in carbonate decomposition-related mass loss in the mineralized groups relative to conventional curing and should not be interpreted as direct measurements of net CO_2_ uptake at the reactor scale.

Notably, the TGA-derived CO_2_-equivalent amount associated with carbonate decomposition was slightly lower for G1 than for G0, whereas its 28 d compressive strength increased from 14.32 to 21.07 MPa. This finding indicates that the higher 28 d strength of the 1% GGF-modified material cannot be attributed simply to a greater amount of carbonate formation. In conjunction with the subsequent FTIR, MIP, and SEM-EDS results, local particle–matrix interactions may contribute to the observed mechanical response; however, the present data do not directly establish a distinct carbonation-mitigation mechanism.

### 3.3. Chemical Bond and Silicate Structure Changes (FTIR)

**Figure 6:**
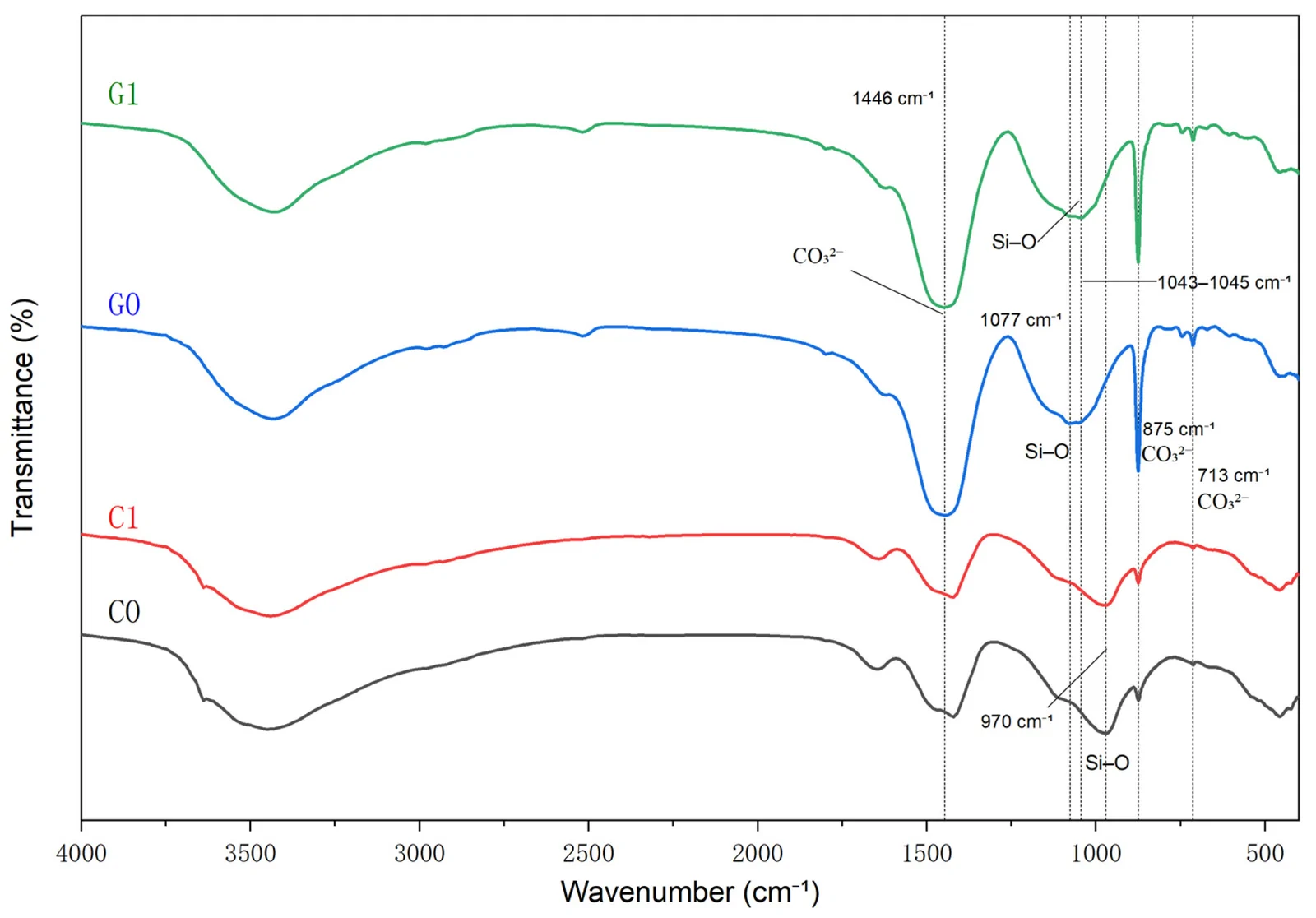
Fourier transform infrared spectra of different specimens.

To further investigate the evolution of the gel structure under CO_2_ exposure, FTIR spectroscopy was performed on the C0, C1, G0, and G1 groups. The FTIR spectra of the different specimens are shown in Figure 6.

As shown in Figure 6, characteristic absorption bands associated with Si–O vibrations in the C–S–H phase were observed at approximately 970 cm^−1^ in the C0 and C1 groups. After mineralization curing, the corresponding absorption band of G0 shifted to 1077 cm^−1^, whereas that of G1 was located at approximately 1043–1045 cm^−1^. The shift of the Si–O-related absorption band toward higher wavenumbers indicates a pronounced change in the silicate network structure, which can be associated with an increased degree of silicate polymerization following C–S–H decalcification [24,25].

Meanwhile, the mineralized specimens exhibited the appearance or enhancement of characteristic absorption bands associated with CO_3_^2−^ at approximately 1446, 875, and 713 cm^−1^. These features are consistent with the XRD and TGA results, indicating a substantial increase in carbonate formation [26]. Overall, prolonged CO_2_ exposure not only promoted CaCO_3_ formation but also induced the structural evolution of C–S–H toward a more highly polymerized silicate network. This process may be accompanied by gel-scale shrinkage and may provide a microstructural basis for subsequent pore coarsening and strength decline.

### 3.4. Pore Structure Evolution (MIP)

The macroscopic mechanical performance of cementitious backfill materials is closely related to their internal pore structure [27]. Meanwhile, curing conditions are important factors governing the porosity and pore-size distribution of cement-based materials [28]. To clarify the physical structural basis of the strength decline associated with prolonged carbonation, mercury intrusion porosimetry (MIP) was employed to quantitatively characterize the pore structure of the C1, G0, and G1 specimens at 28 d.

**Figure 7:**
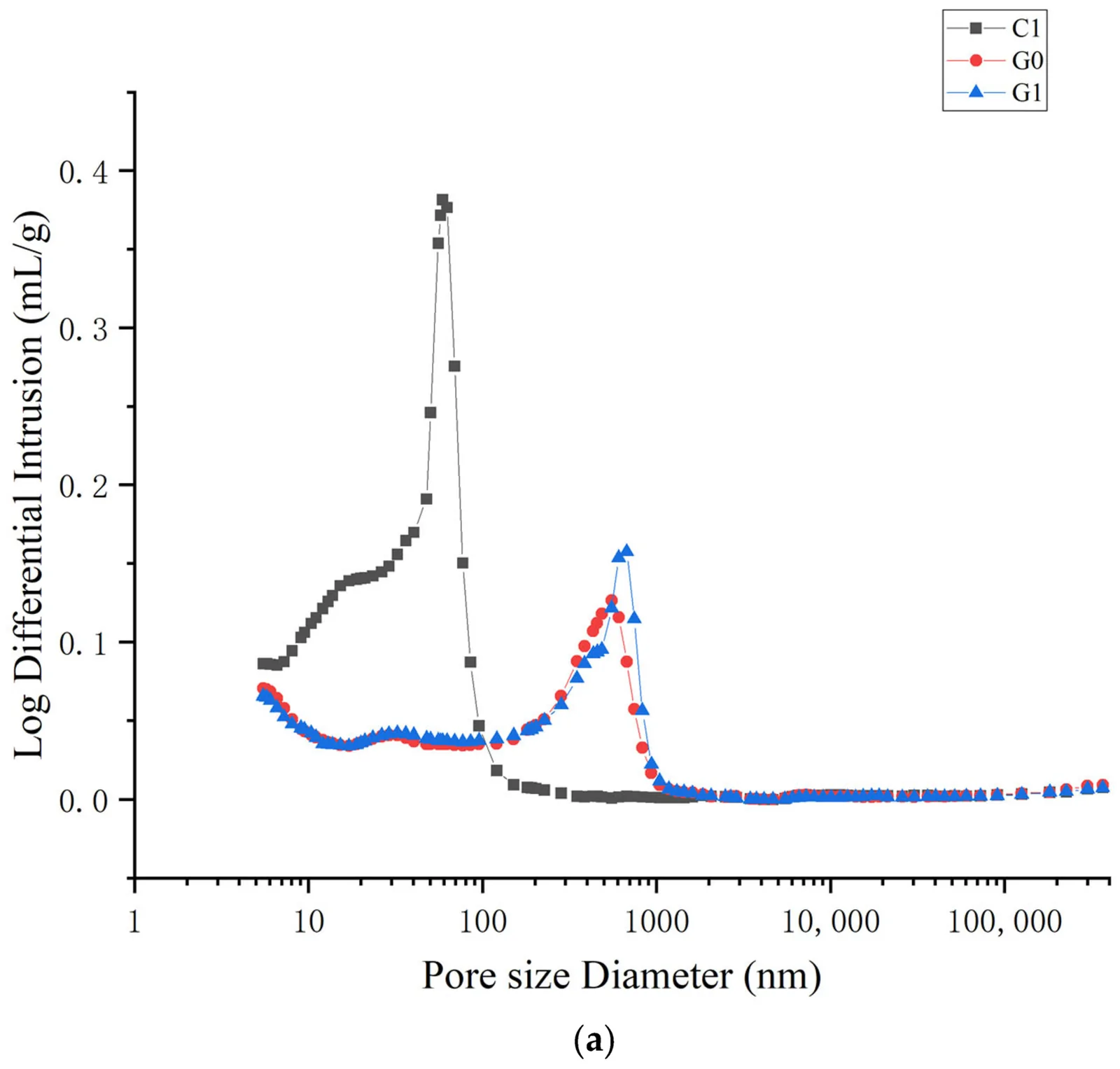

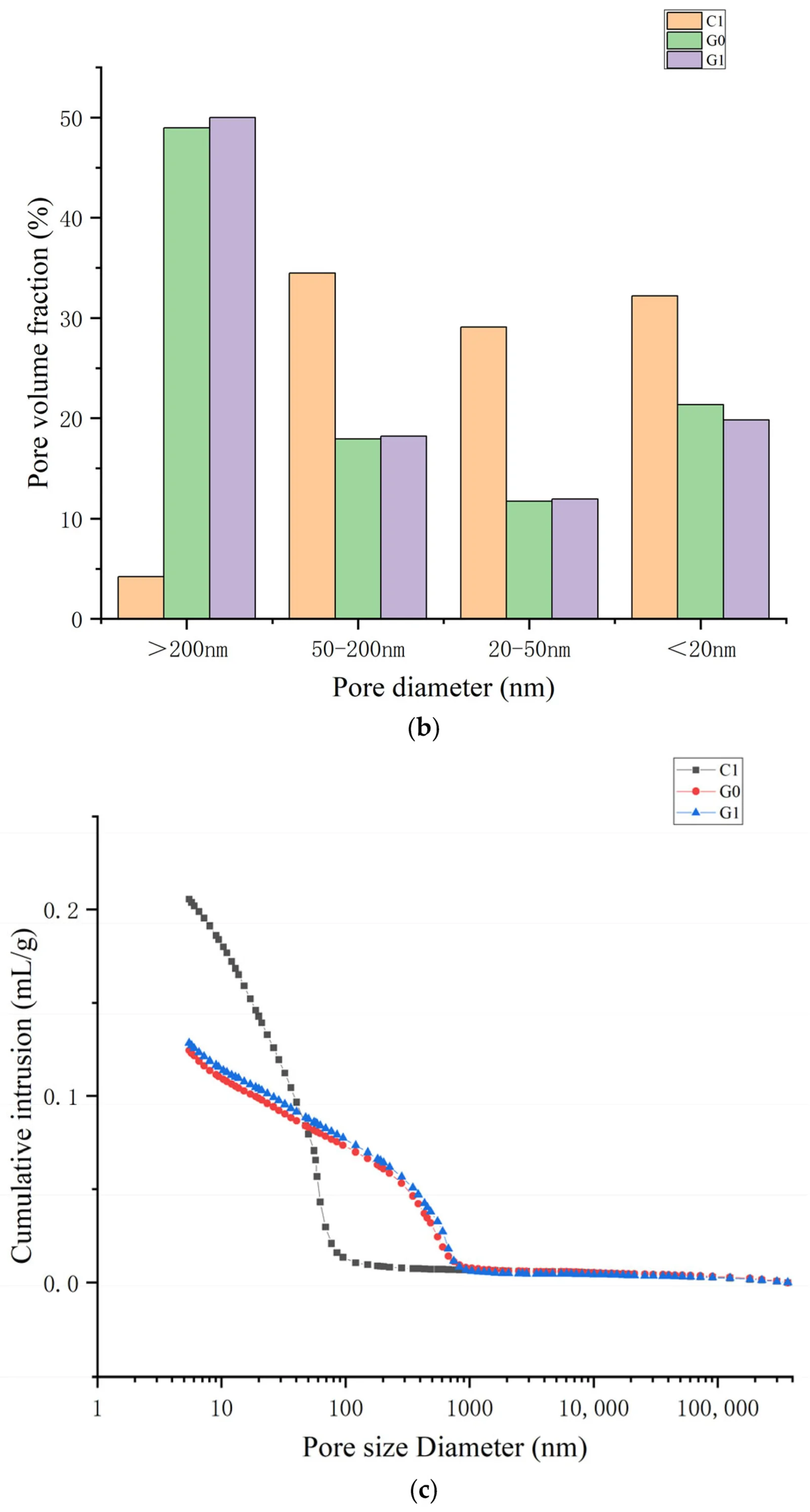
MIP pore structure characteristics of different specimens: (**a**) Pore size distribution; (**b**) Pore volume fraction; (**c**) Cumulative pore volume.

Figure 7a–c show the pore-size distribution, pore-volume fraction, and cumulative pore volume of the different specimens, respectively. The results show that the conventionally hydrated C1 group had a total porosity of 30.96% and a median pore diameter (by volume) of only 37.15 nm. According to the pore-hazard classification proposed by Wu Zhongwei, pores smaller than 20 nm are classified as harmless pores, those ranging from 20 to 50 nm as less harmful pores, those from 50 to 200 nm as harmful pores, and those larger than 200 nm as very harmful pores [29]. The C1 group was dominated by harmless and less harmful pores, indicating a relatively dense and structurally favorable pore system.

However, after 28 d of prolonged high-concentration CO_2_ mineralization curing, the pore structure underwent pronounced evolution. The total porosities of G0 and G1 decreased to 21.32% and 21.86%, respectively, suggesting that carbonate formation contributed to the filling of part of the original pore space. Nevertheless, the reduction in total porosity was not accompanied by an overall refinement of pore size. The median pore diameters of G0 and G1 increased markedly to 190.79 and 202.72 nm, respectively, approaching the critical size of very harmful pores. These results indicate the coexistence of a reduction in total pore volume and an increase in relatively large pores or cracks.

This evolution suggests that CaCO_3_ formed during CO_2_ mineralization may fill part of the existing capillary pores, whereas C–S–H decalcification and the associated shrinkage may contribute to local pore coarsening. Notably, the median pore diameter of G1 was slightly larger than that of G0, while its 28 d compressive strength was substantially higher. This apparent decoupling between median pore diameter and compressive strength indicates that overall pore-size refinement alone cannot explain the improved mechanical performance of G1. Local particle–matrix interfacial characteristics may also contribute to the mechanical response [30]; however, their specific roles cannot be determined from MIP data alone. The SEM observations in Section 3.5 are therefore used only to explore possible local morphological explanations rather than to demonstrate a specific crack-obstruction mechanism.

It should be noted that pore-size parameters obtained by MIP can be affected by sample pretreatment, pore connectivity, and the ink-bottle effect. Therefore, the median pore diameter reported in this study is primarily used for relative comparison of pore-structure characteristics among the specimens and should not be directly interpreted as the true geometric pore size of the material [31,32].

### 3.5. Microstructural Morphology and Local Interfacial Characteristics (SEM-EDS)

**Figure 8:**
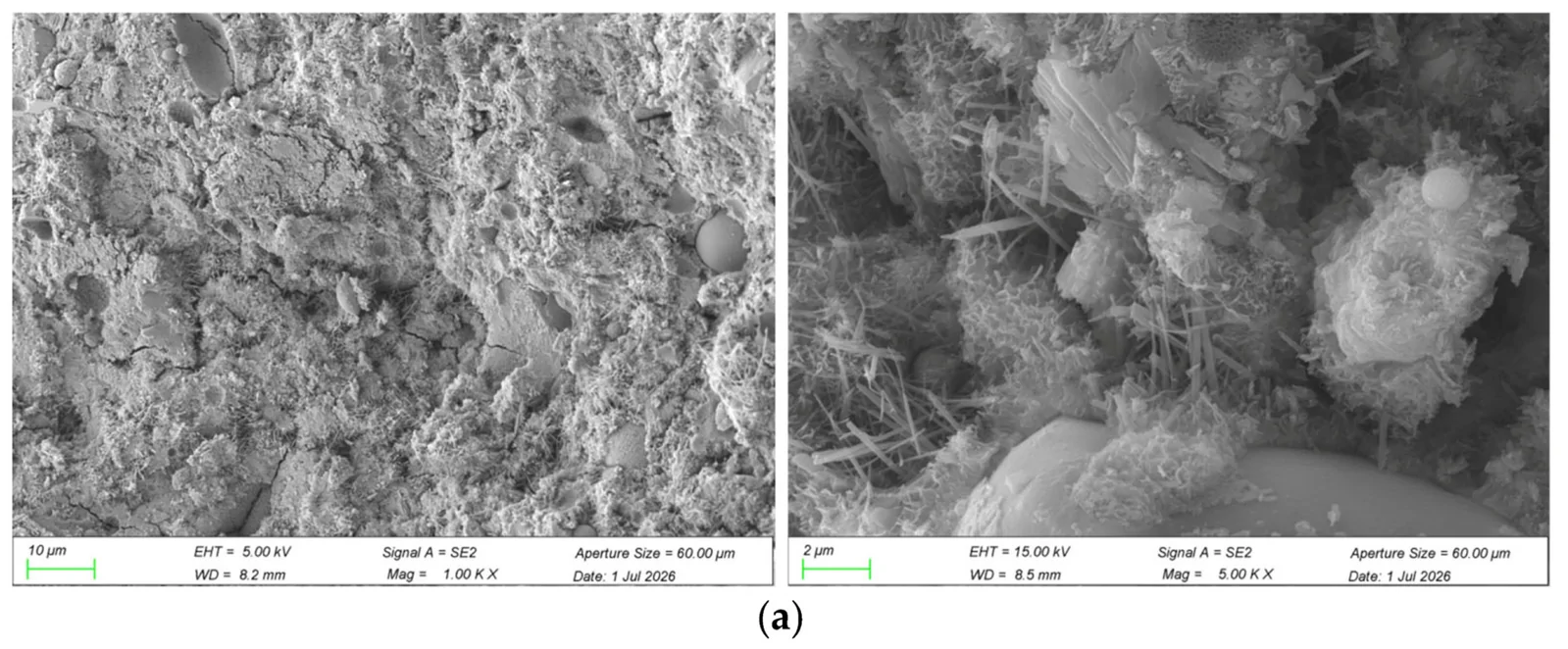

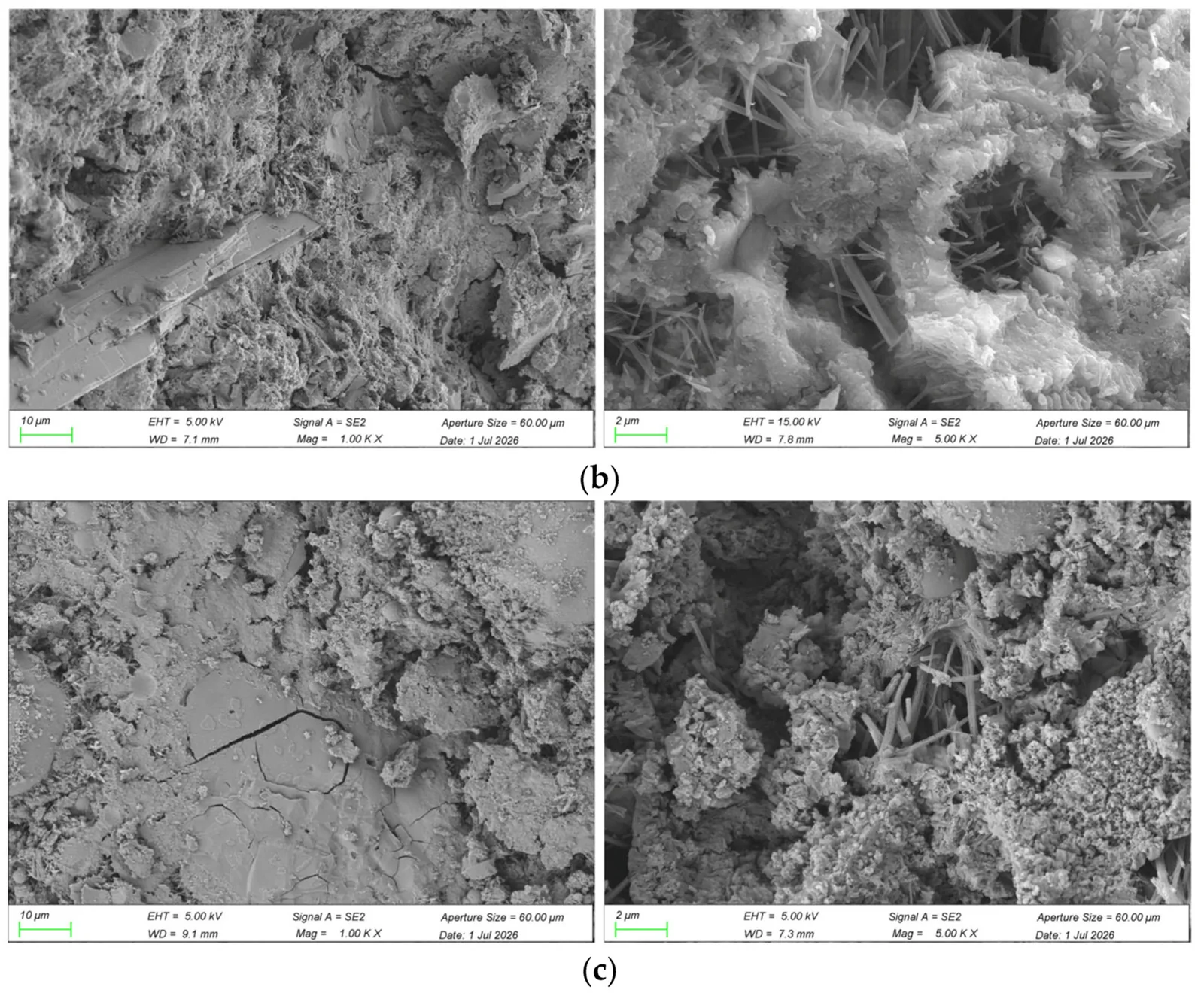
SEM microscopic morphology evolution of different specimens at 28 d: (**a**) C1; (**b**) G1; (**c**) G0.

**Figure 9:**
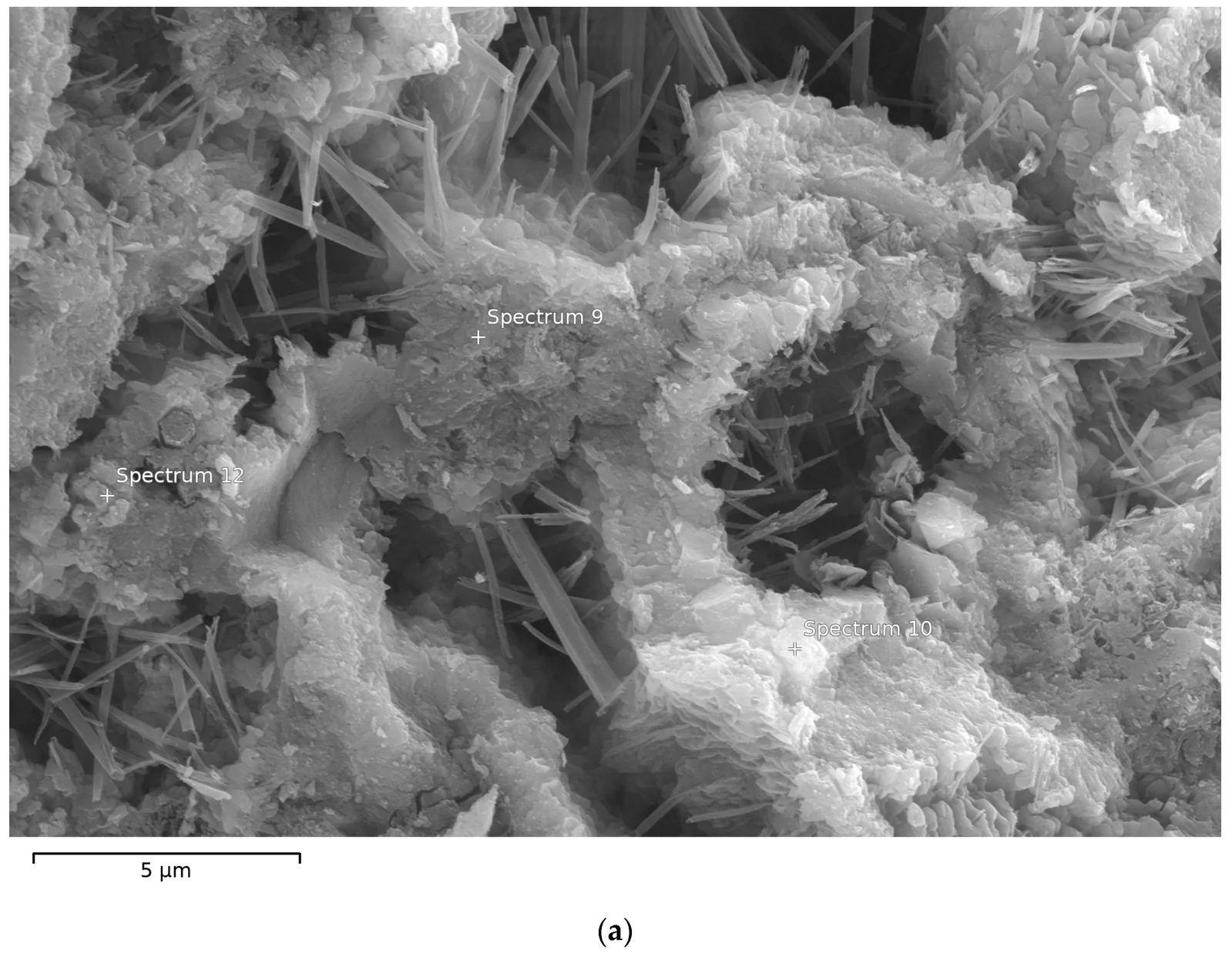

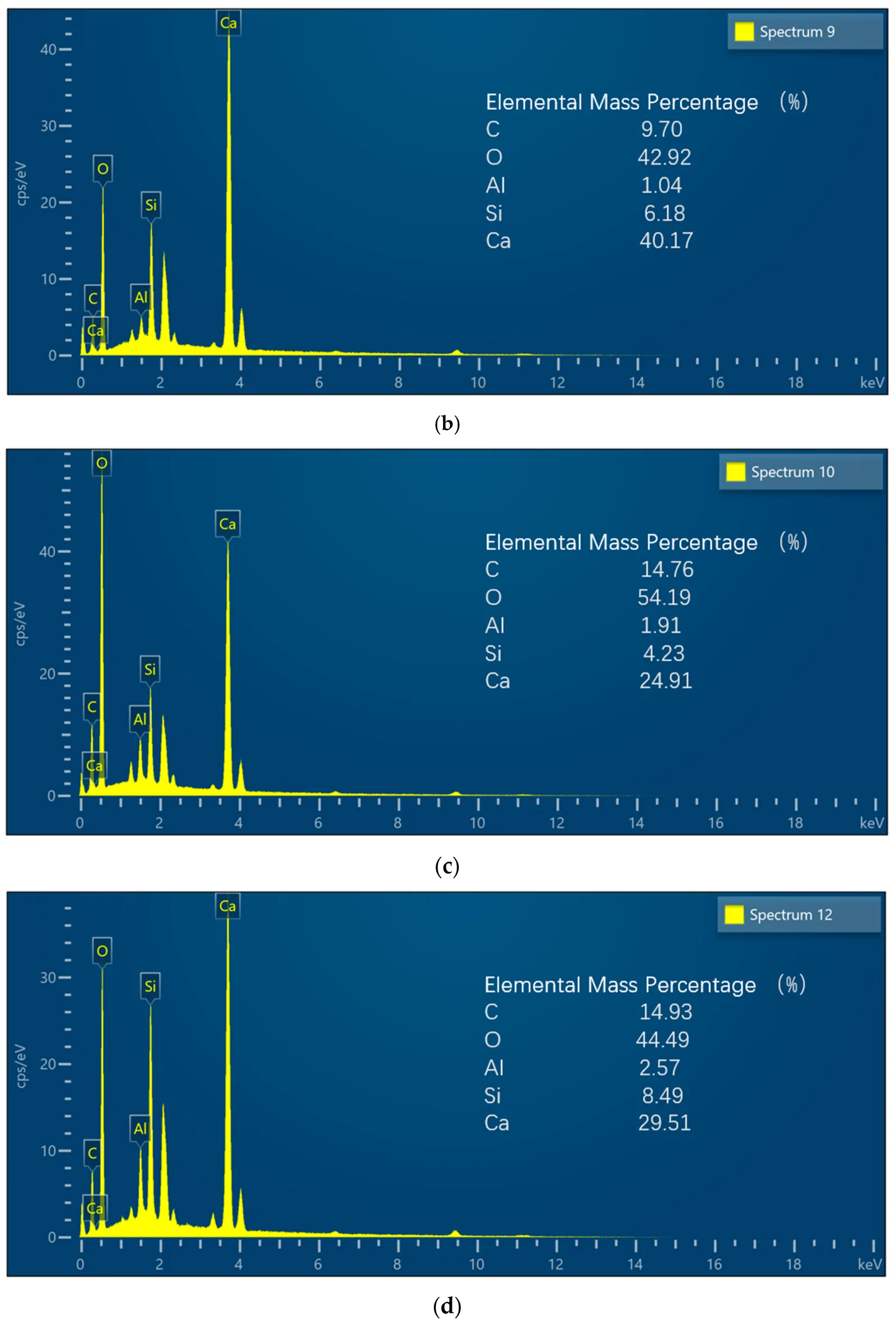
SEM-EDS analysis of selected local regions in the G1 specimen: (**a**) locations of EDS analyses; (**b**) EDS spectrum of Spectrum 9; (**c**) EDS spectrum of Spectrum 10; (**d**) EDS spectrum of Spectrum 12.

To further examine the local morphological and compositional characteristics associated with the preceding results, SEM-EDS characterization was performed on the fracture surfaces of the specimens at 28 d. Figure 8 compares C1, G1, and G0 at two magnifications, with attention to matrix-rich regions, particle-associated regions, and visible cavities and discontinuities in each specimen. The C1–G1 comparison examines different curing regimes at the same GGF dosage, whereas the G0–G1 comparison examines the effect of GGF addition under CO_2_ mineralization curing. Given the relevance of interfacial regions to the properties of cementitious materials [33,34], particle–matrix contacts were also examined. Figure 9 provides localized semiquantitative elemental information for three positions in G1, designated as Spectra 9, 10, and 12. These results are interpreted together with the bulk characterization results rather than used for direct identification of individual phases.

As shown in Figure 8a, the conventionally hydrated C1 specimen exhibits a heterogeneous fracture surface comprising fine matrix material, partially exposed particles, and irregular cavities. In the low-magnification image, granular and flocculent material covers substantial portions of the exposed solids, while several rounded particles retain relatively smooth surfaces. These rounded features are consistent with residual fly ash particles embedded in the hydrated matrix. The high-magnification image further reveals surface deposits and needle-like features extending into spaces between adjacent solids. Thus, the C1 microstructure contains both regions with extensive matrix coverage and relatively open particle-associated regions. This spatial variation should be considered when interpreting the apparent compactness of individual areas.

The mineralized G0 specimen in Figure 8c displays a different arrangement of exposed solids and surrounding matrix material. The low-magnification image contains a broad, relatively smooth domain intersected by a pronounced crack-like discontinuity, together with rough particulate regions and gaps along their boundaries. At higher magnification, granular deposits cover irregular aggregates, whereas open cavities remain between neighboring aggregates and contain needle-like features. Product-covered surfaces therefore coexist with local discontinuities and incompletely filled spaces. Considered alongside the XRD, FTIR, and TGA results, this morphology is compatible with heterogeneous microstructural rearrangement during CO_2_ mineralization, in which carbonate formation accompanies changes in the hydration products. However, because the specimens were fractured and vacuum-dried, the observed discontinuities cannot be assigned exclusively to pre-existing mineralization-induced damage.

For G1, the low-magnification image in Figure 8b shows a prominent elongated inclusion with a relatively smooth exposed face and adhering fine material along parts of its edges. Its morphology is compatible with a ground fiber-derived fragment, although its identity cannot be established from morphology alone. Adjacent regions contain rough matrix material, partially exposed particles, and visible cavities. The high-magnification image reveals irregular aggregates covered by fine granular and plate-like deposits, with needle-like features extending into the intervening spaces. These observations indicate localized contact between particles and surrounding matrix material, while also demonstrating that open regions persist. Across the three specimens, surface deposits, needle-like features, and cavities are recurring morphological features; their occurrence alone therefore does not distinguish GGF modification. The comparison instead concerns their local arrangement and association with exposed particles and matrix discontinuities, rather than a uniform change in overall compactness.

The EDS results in Figure 9 provide complementary information from three spatially separated positions within the displayed G1 field. Spectrum 9, located on the central irregular aggregate, contains 40.17 wt% Ca, 42.92 wt% O, and 9.70 wt% C, together with 6.18 wt% Si and 1.04 wt% Al. This Ca-rich composition is consistent with a carbonate-associated region when interpreted alongside the bulk XRD and TGA results, while the Si and Al signals indicate contributions from additional constituents within the analyzed volume. Spectrum 10, located on the lower-right aggregate, also contains predominantly C, O, and Ca, with contents of 14.76, 54.19, and 24.91 wt%, respectively, together with 4.23 wt% Si and 1.91 wt% Al. Although its normalized Ca content is lower than that of Spectrum 9, this difference alone cannot establish a lower carbonate abundance.

Spectrum 12, located in the left-hand particle-associated region, contains 29.51 wt% Ca, 44.49 wt% O, and 14.93 wt% C, together with 8.49 wt% Si and 2.57 wt% Al. Its higher Si and Al contents indicate a greater relative contribution from Si- and Al-bearing constituents than at the other two positions. The atomic Ca/Si ratios, calculated from the reported atomic fractions, are approximately 4.56, 4.12, and 2.44 for Spectra 9, 10, and 12, respectively. These ratios characterize the local analyzed volumes and should not be interpreted as the stoichiometry of a single hydrate phase. Collectively, the three measurements show that the association of C, O, and Ca is not confined to Spectrum 9, while the varying Si and Al contributions demonstrate local compositional heterogeneity. They support an interpretation involving carbonate-associated regions with variable contributions from silicate- and aluminosilicate-bearing constituents, rather than a chemically uniform matrix.

At 28 d, G1 exhibited a compressive strength of 21.07 MPa, compared with 14.32 MPa for G0, despite its slightly higher total porosity and larger MIP-derived median pore diameter. The strength difference was therefore not accompanied by a reduction in either bulk pore metric. G0 and G1 nevertheless differed in their thermal and silicate-related spectral responses. Their mass losses within 50–300 °C were 7.32% and 4.02%, respectively, while their Si–O-related FTIR bands occurred at approximately 1077 and 1043–1045 cm^−1^. These observations identify differences in low-temperature mass-loss behavior and silicate-related spectral characteristics that are not captured by total porosity or median pore diameter. The lower low-temperature mass loss of C1 relative to C0 further shows that the thermal differences associated with GGF incorporation were not exclusive to CO_2_ mineralization curing, consistent with the observation that GGF also increased strength under conventional hydration curing.

The strength response is therefore interpreted by considering the characteristics of the hydrated matrix together with its pore structure. The phase and spectral changes discussed in Section 3.2 and Section 3.3 are consistent with carbonate formation occurring alongside alteration of silicate-bearing hydrates. This interpretation accommodates the coexistence of lower total porosity and lower mechanical strength: in the matched-dosage C1–G1 comparison, the mineralized specimen exhibited lower total porosity but a larger median pore diameter and lower 28 d strength. For G0 and G1, the thermal and spectral differences likewise identify aspects of the cured material that an explanation based solely on bulk densification would overlook. The SEM–EDS observations provide complementary spatial information through the product-covered particle surfaces, local matrix contacts, and compositional heterogeneity within the examined G1 field. Together, these results establish that the strength advantage of G1 was accompanied by differences in the characteristics of the cured matrix, without a corresponding reduction in the two bulk pore metrics.

The present study investigated only one GGF source, containing 52.43 wt% CaO, 45.89 wt% SiO_2_, 0.08 wt% Na_2_O, and 0.12 wt% Al_2_O_3_. Variations in glass composition may affect dissolution, interfacial reactions, and carbonate formation under CO_2_ curing [35]. Therefore, the favorable performance observed for 1% GGF should be considered specific to the material composition and curing conditions investigated here, and its applicability to GGF of different compositions requires further verification.

## 4. Conclusions

(1) Prolonged high-concentration CO_2_ mineralization curing significantly altered the strength evolution of the fly ash–cement backfill material. The compressive strength of the G0 group reached 22.42 MPa at 7 d but decreased to 14.32 MPa at 28 d, corresponding to a reduction of approximately 36.1% from the 7 d peak and indicating pronounced late-age strength decline. After incorporating 1% GGF, the compressive strengths of the G1 group at 7 and 28 d were 27.09 and 21.07 MPa, respectively, and the 28 d strength was approximately 47.1% higher than that of G0. These results show that 1% GGF improved 28 d strength and reduced the extent of 7–28 d strength decline under the investigated prolonged CO_2_ mineralization condition. Within the investigated dosage range of 0–2%, 1% GGF exhibited the most favorable overall mechanical performance.

(2) CO_2_ mineralization promoted the consumption of Ca(OH)_2_ and the formation of carbonate phases. XRD showed that the characteristic diffraction peaks of Ca(OH)_2_ were markedly weakened or disappeared after mineralization, accompanied by the intensification of carbonate-related diffraction features near 2θ ≈ 29.3°. TGA further showed that the mass loss associated with carbonate decomposition increased from 4.39% in C1 to 15.36% in G1 within the 600–800 °C range, confirming substantial carbonate formation after 28 d of mineralization curing.

(3) Prolonged CO_2_ exposure resulted in the coexistence of reduced total porosity and increased median pore diameter. FTIR analysis showed that the Si–O-related band of G0 shifted from approximately 970 cm^−1^ to 1076.57 cm^−1^, indicating pronounced structural evolution of the C–S–H phase. MIP measurements showed that the median pore diameters of C1, G0, and G1 were 37.15, 190.79, and 202.72 nm, respectively. These results indicate that carbonate formation can partially fill existing pores while C–S–H decalcification and associated shrinkage may simultaneously promote local pore coalescence, coarsening, and defect connectivity. This structural evolution is consistent with the late-age strength decline observed in G0.

(4) The higher 28 d compressive strength of G1 compared with G0 was not accompanied by greater carbonate-related TGA mass loss, lower total porosity, or a smaller MIP-derived median pore diameter. Instead, differences in low-temperature mass loss and Si–O-related FTIR band positions were observed between the two mineralized mixtures, indicating distinct thermal and silicate-related spectral responses. SEM observations further revealed local morphological variations in particle-associated regions, while EDS demonstrated compositional heterogeneity within the examined G1 regions. These results establish an association between the mechanical response and multiple microstructural characteristics, but do not directly identify the processes responsible for the strength enhancement. Within the investigated dosage range, 1% GGF achieved the highest absolute 28 d compressive strength under CO_2_ mineralization curing and exhibited a smaller 7–28 d strength decline than the unmodified mixture.

## Figures and Tables

**Table 1:** Performance parameters of Portland cement.

Material	Chloride Ion (%)	Specific Surface Area (m^2^/kg)	Loss on Ignition (%)	3 d Compressive Strength (MPa)	Initial Setting Time (Min)	Final Setting Time (Min)
Cement	0.044	340	3.09	30.8	202	251

**Table 2:** Chemical compositions of the raw materials (wt%).

Material	SiO_2_	Al_2_O_3_	Fe_2_O_3_	CaO	MgO	SO_3_	K_2_O	Na_2_O	TiO_2_
Cement	20.80	4.80	3.20	60.10	4.29	2.52	0.60	0.15	0.35
Fly ash	53.36	29.09	4.87	3.27	1.15	0.85	1.12	0.45	1.25
Ground glass fiber	45.89	0.12	0.42	52.43	0.56	0.02	0.02	0.08	0.08

**Table 3:** Mix proportions of the backfill materials (parts by mass per 100 parts of base binder).

Group	Curing Regime	Cement	Fly Ash	GGF *	Water
C0	Conventional	70.0	30.0	0.0	45.0
C1	Conventional	70.0	30.0	1.0	45.0
C2	Conventional	70.0	30.0	2.0	45.0
G0	CO_2_ Mineralization	70.0	30.0	0.0	45.0
G1	CO_2_ Mineralization	70.0	30.0	1.0	45.0
G2	CO_2_ Mineralization	70.0	30.0	2.0	45.0

* GGF contents of 1 and 2 parts correspond to approximately 0.99 and 1.96 wt.% of the total dry solids, respectively.

## Data Availability

The original contributions presented in this study are included in the article. Further inquiries can be directed to the corresponding author.
